# Supplementary material for: Quantifying Population Reversibility of Sensor Performance in Multi‐Cycle Single‐Sensor Recovery Assay
Source: Small. 2026 Apr 14;22(33):e73339. doi: 10.1002/smll.73339 (PMC13262247; doi:10.1002/smll.73339)
Supplement: Supplementary file 1 — Supporting File 1: smll73339‐sup‐0001‐SuppMat.pdf. [file SMLL-22-e73339-s003.pdf]

Supplemental information

Quantifying Population Reversibility of Sensor Performance in Multi-Cycle Single-Sensor Recovery Assay

Geffen Rosenberg[a] and Gili Bisker[a,b,c,d,e,f,*]

[a] School of Biomedical Engineering, Faculty of Engineering, Tel Aviv University, Tel Aviv 6997801, Israel

[b] Center for Physics and Chemistry of Living Systems, Tel Aviv University, Tel Aviv 6997801, Israel

[c] Center for Nanoscience and Nanotechnology, Tel Aviv University, Tel Aviv 6997801, Israel

[d] Center for Light-Matter Interaction, Tel Aviv University, Tel Aviv 6997801, Israel

[e] Sagol School of Neuroscience, Tel Aviv University, Tel Aviv 6997801, Israel

[f] The Center for Computational Molecular and Materials Science, Tel Aviv University, Tel Aviv 6997801, Israel

[*] Email: bisker@tauex.tau.ac.il

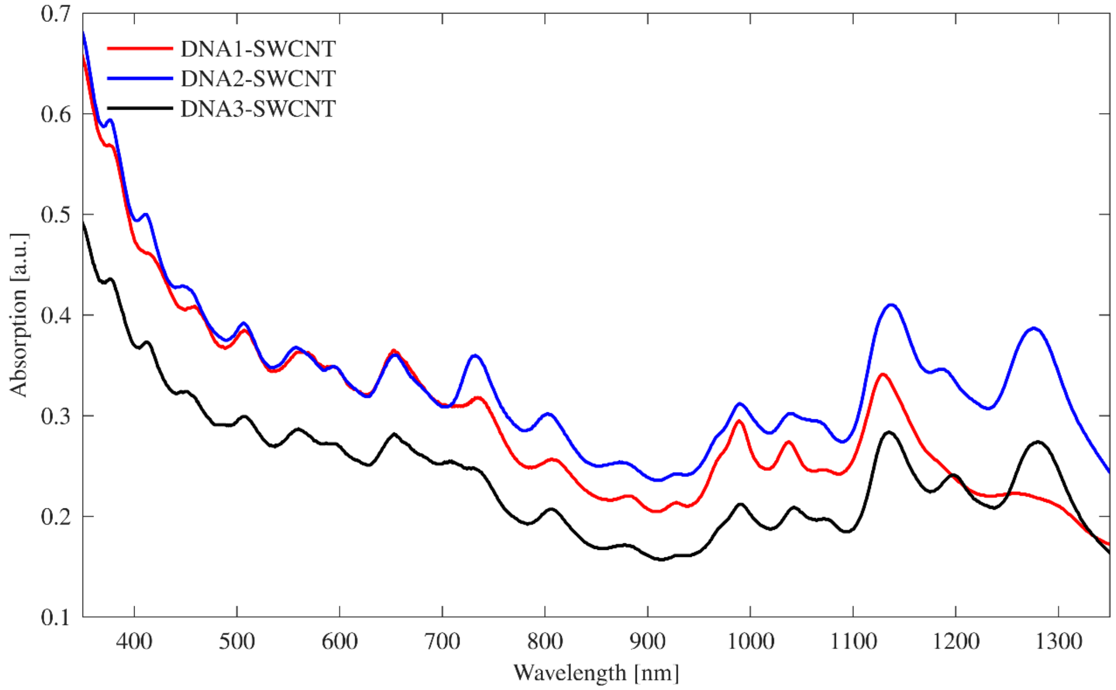

Figure S1. Absorption spectrum of ssDNA functionalized SWCNTs.

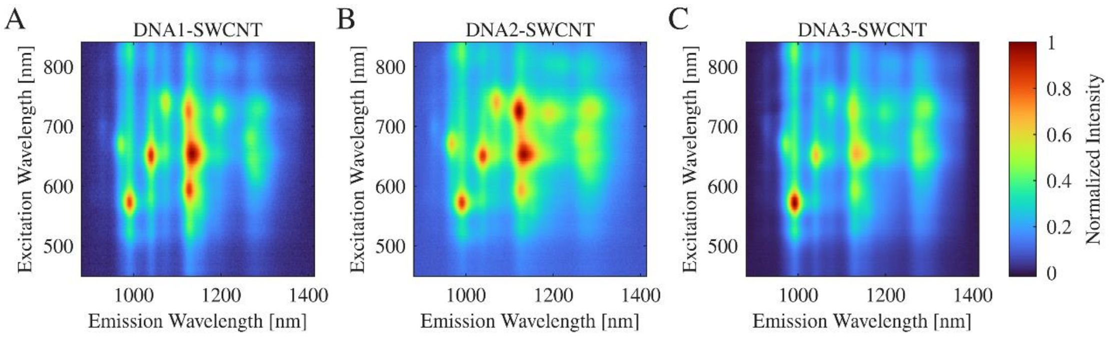

Figure S2. Excitation-emission maps normalized to the maximum emission intensity of ssDNA functionalized SWCNT. (A) DNA1-SWCNT. (B) DNA2-SWCNT. (C) DNA3-SWCNT

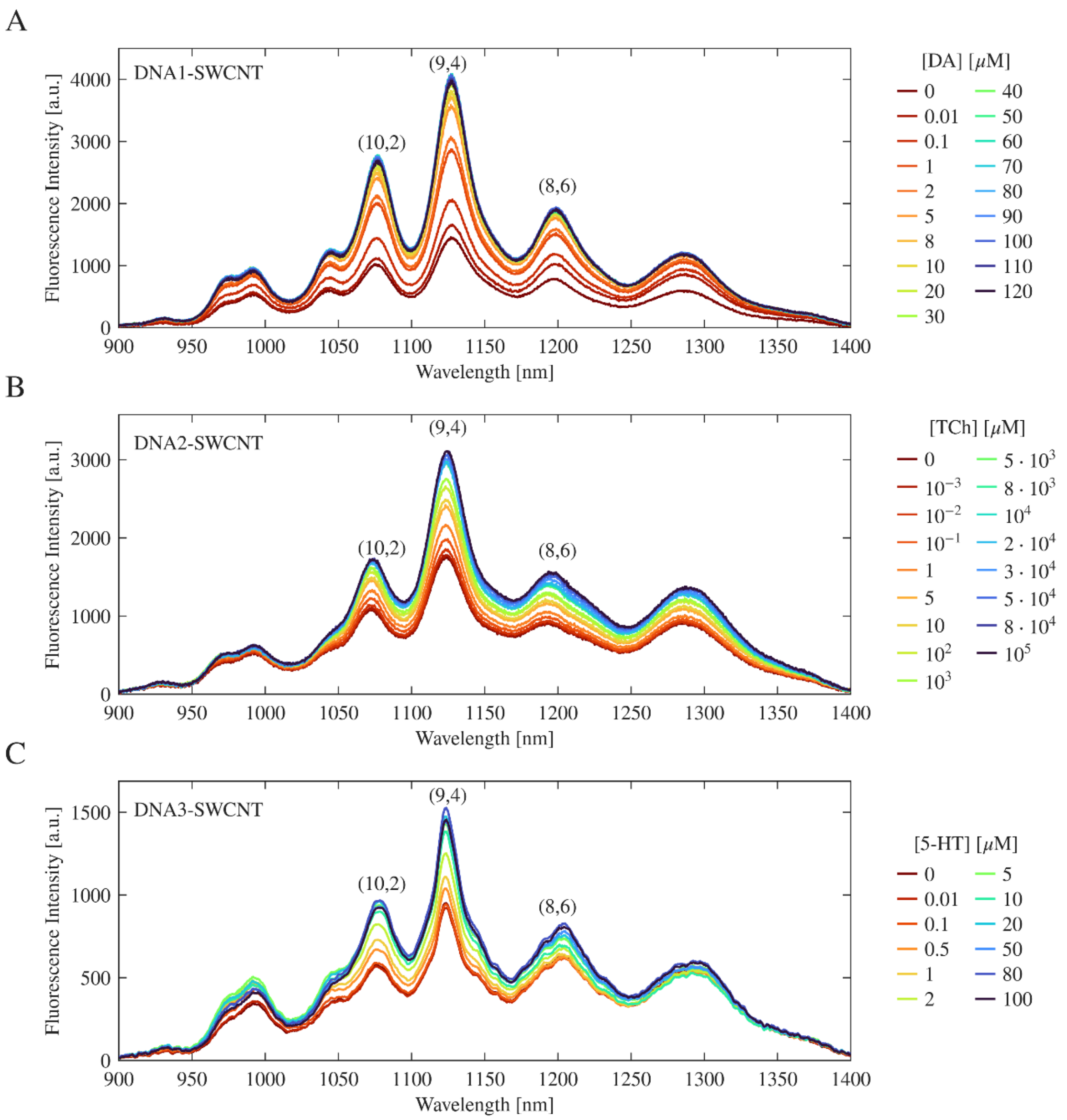

Figure S3. Fluorescence spectra of ssDNA functionalized SWCNT upon the introduction of increasing concentrations of analyte. (A) DNA1-SWCNT with the introduction of DA. (B) DNA2-SWCNT with the introduction of TCh. (C) DNA3-SWCNT with the introduction of 5-HT. Chirality peaks of (10,2), (9,4), and (8,6) chiralities are marked.

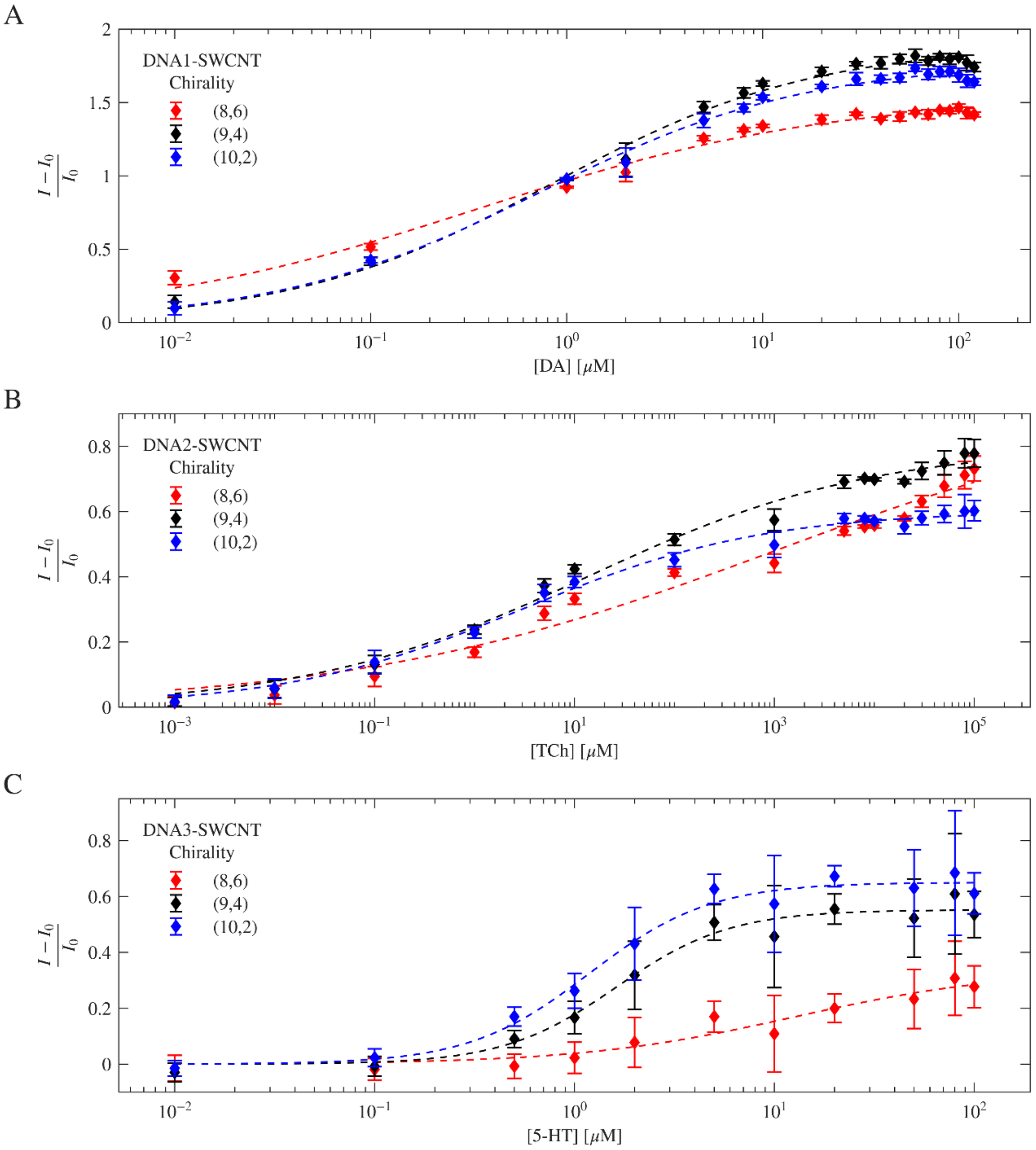

Figure S4. Calibration of ssDNA-functionalized SWCNT normalized fluorescence response of different SWCNT chiralities to the corresponding analyte, where the data points represent experimental data ($n = 3$), and the dashed line represents the calibration fit according to the Hill equation. (A) DNA1-SWCNTs response to DA. (B) DNA2-SWCNTs response to TCh. (C) DNA3-SWCNTs response to 5-HT.

Table S1. Calibration fit parameters and their 95% confidence intervals. LOD values were calculated using the calibration curve.

	Chirality	β	K [μM]	n	LOD [μM]
**DNA1-SWCNT**	(8,6)	1.55 (1.48, 1.62)	0.352 (0.23, 0.47)	0.481 (0.4, 0.56)	0.002
	(9,4)	1.895 (1.83, 1.96)	0.842 (0.65, 1.03)	0.652 (0.56, 0.75)	0.013
	(10,2)	1.777 (1.72, 1.83)	0.733 (0.58, 0.73)	0.641 (0.56, 0.73)	0.011
**DNA2-SWCNT**	(8,6)	0.958 (0.54, 1.38)	1001 (0, 5968)	0.205 (0.12, 0.29)	0.022
	(9,4)	0.801 (0.74, 0.86)	13.58 (1.6, 25.57)	0.305 (0.24, 0.37)	0.012
	(10,2)	0.603 (0.58, 0.63)	3.04 (1.58, 4.5)	0.361 (0.3, 0.42)	0.048
**DNA3-SWCNT**	(8,6)	0.339 (0.13, 0.55)	12.65 (-13.77, 39.07)	0.807 (0.06, 1.55)	5.315
	(9,4)	0.552 (0.51, 0.6)	1.622 (1.1, 2.14)	1.532 (0.84, 2.23)	0.582
	(10,2)	0.65 (0.61, 0.69)	1.19 (0.88, 1.5)	1.437 (0.89, 1.98)	0.318

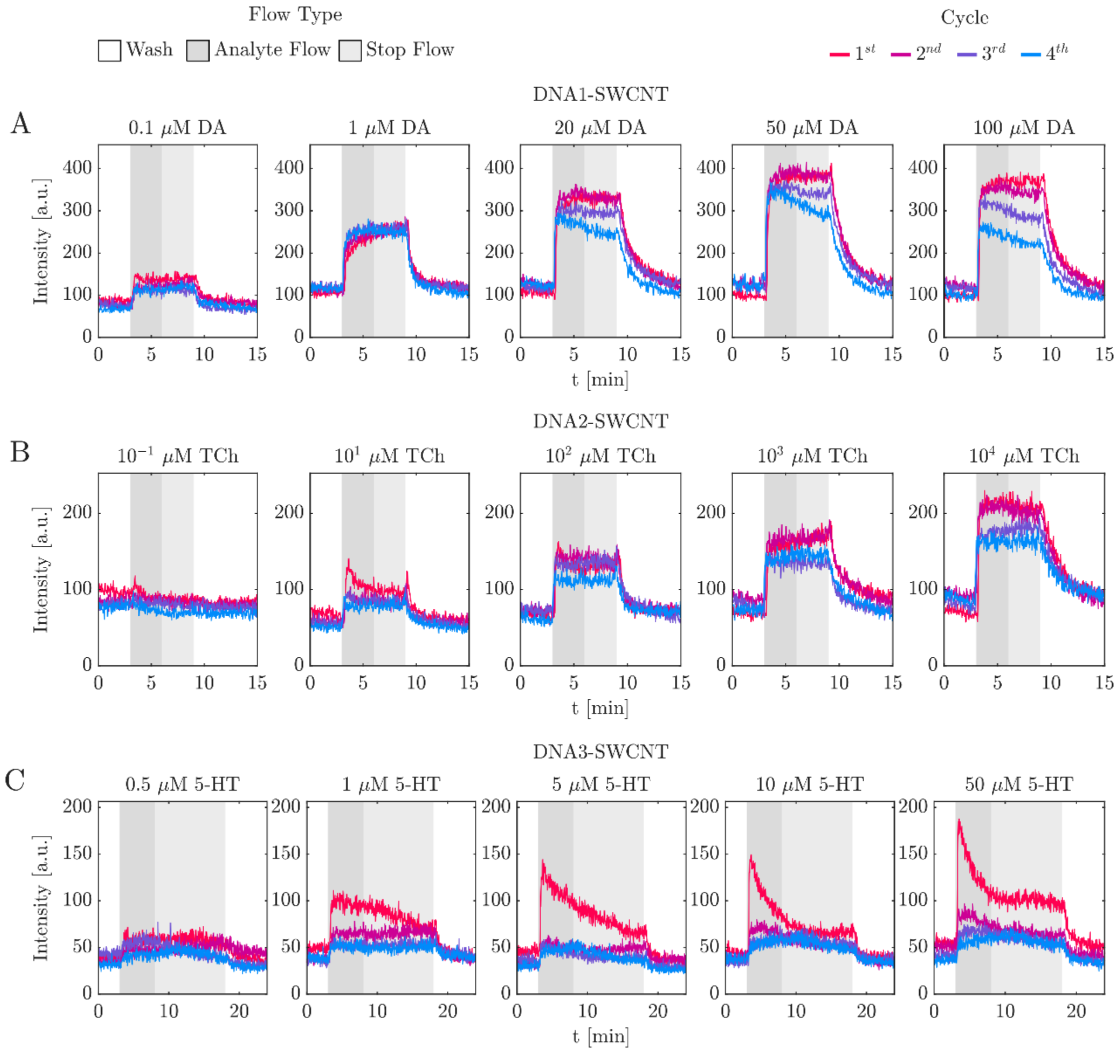

Figure S5. Comparison of the mean measured fluorescence value of the FOV between cycles. White background represents PBS flow, dark grey represents analyte flow, and light grey represents the stop of the pump's flow. (A) DNA1-SWCNT flow experiments with DA as an analyte. (B) DNA2-SWCNT flow experiments with TCh as analyte. (C) DNA3-SWCNT flow experiments with 5-HT as analyte.

Table S2. Mean FOV I_0 and I values for each SWCNT sensor, analyte concentration, and cycle.

	**[Analyte] [µM]**		**1st Cycle**	**2nd Cycle**	**3rd Cycle**	**4th Cycle**
**DNA1-SWCNT**	0.1	I_0	85.8 ± 5.5	75.2 ± 6.1	73.1 ± 5.7	69.0 ± 5.8
		I	139.5 ± 6.3	117.0 ± 8.0	109.9 ± 4.9	113.4 ± 7.3
	1	I_0	109.1 ± 6.1	112.1 ± 6.7	119.9 ± 12.8	119.7 ± 18.3
		I	222.4 ± 11.2	234.4 ± 7.5	252.3 ± 7.5	249.3 ± 7.8
	20	I_0	105.6 ± 6.3	123.1 ± 6.3	114.8 ± 7.2	122.4 ± 13.5
		I	308.4 ± 10.9	331.0 ± 7.6	300.5 ± 9.5	277.5 ± 9.8
	50	I_0	96.3 ± 4.7	121.5 ± 8.3	122.9 ± 18.5	134.7 ± 33.7
		I	358.8 ± 14.6	381.2 ± 10.9	354.5 ± 8.4	342.1 ± 9.6
	100	I_0	99.0 ± 5.5	119.1 ± 5.2	115.1 ± 5.7	102.2 ± 19.3
		I	354.3 ± 10.9	349.7 ± 6.9	316.0 ± 7.7	257.5 ± 9.6
**DNA2-SWCNT**	0.1	I_0	94.8 ± 4.9	84.1 ± 5.3	82.7 ± 4.0	79.4 ± 4.3
		I	96.2 ± 6.1	87.8 ± 4.0	84.2 ± 4.6	76.8 ± 5.2
	10	I_0	65.7 ± 3.9	56.7 ± 3.5	56.3 ± 4.8	53.9 ± 9.3
		I	118.5 ± 9.6	90.8 ± 4.4	89.1 ± 5.2	78.0 ± 4.1
	10^2	I_0	64.9 ± 4.5	73.1 ± 5.7	75.8 ± 10.7	65.7 ± 16.7
		I	144.5 ± 8.7	141.7 ± 5.6	132.0 ± 5.2	112.2 ± 5.7
	10^3	I_0	68.3 ± 3.5	88.4 ± 11.0	79.9 ± 17.7	83.3 ± 23.6
		I	154.2 ± 6.3	163.5 ± 7.9	138.2 ± 5.9	137.9 ± 6.2
	10^4	I_0	66.8 ± 4.1	97.8 ± 21.6	87.0 ± 25.8	98.5 ± 29.2
		I	209.7 ± 7.8	206.0 ± 7.0	171.5 ± 5.1	163.0 ± 5.9
**DNA3-SWCNT**	0.5	I_0	38.3 ± 3.8	34.1 ± 3.0	44.0 ± 3.9	31.6 ± 2.4
		I	59.3 ± 5.2	45.9 ± 3.3	54.2 ± 3.5	39.5 ± 4.0
	1	I_0	49.7 ± 3.8	38.4 ± 2.5	34.8 ± 3.0	38.0 ± 4.0
		I	99.2 ± 4.9	62.7 ± 5.3	52.7 ± 3.3	51.3 ± 3.2
	5	I_0	46.8 ± 4.1	31.1 ± 2.6	35.9 ± 3.1	30.7 ± 2.8
		I	126.6 ± 6.9	54.3 ± 3.7	51.6 ± 3.0	45.8 ± 4.2
	10	I_0	42.1 ± 3.4	40.4 ± 3.3	34.5 ± 2.5	38.6 ± 3.3
		I	133.8 ± 8.5	68.8 ± 4.2	53.4 ± 4.3	51.5 ± 4.1
	50	I_0	54.4 ± 3.7	51.5 ± 4.1	41.1 ± 3.7	37.3 ± 2.7
		I	167.7 ± 10.7	84.5 ± 4.9	65.4 ± 4.0	53.0 ± 3.9

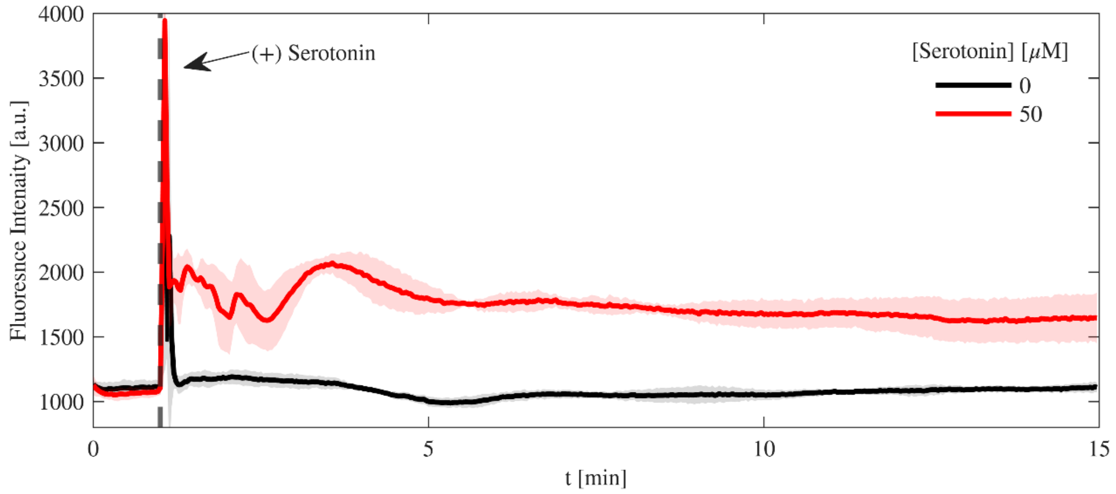

Figure S6. Continuous bulk fluorescence response of the (9,4) chirality of DNA3-SWCNT to 5-HT. The red line represents the fluorescence response to 50 µM 5-HT, while the black line represents the control (0 µM 5-HT).

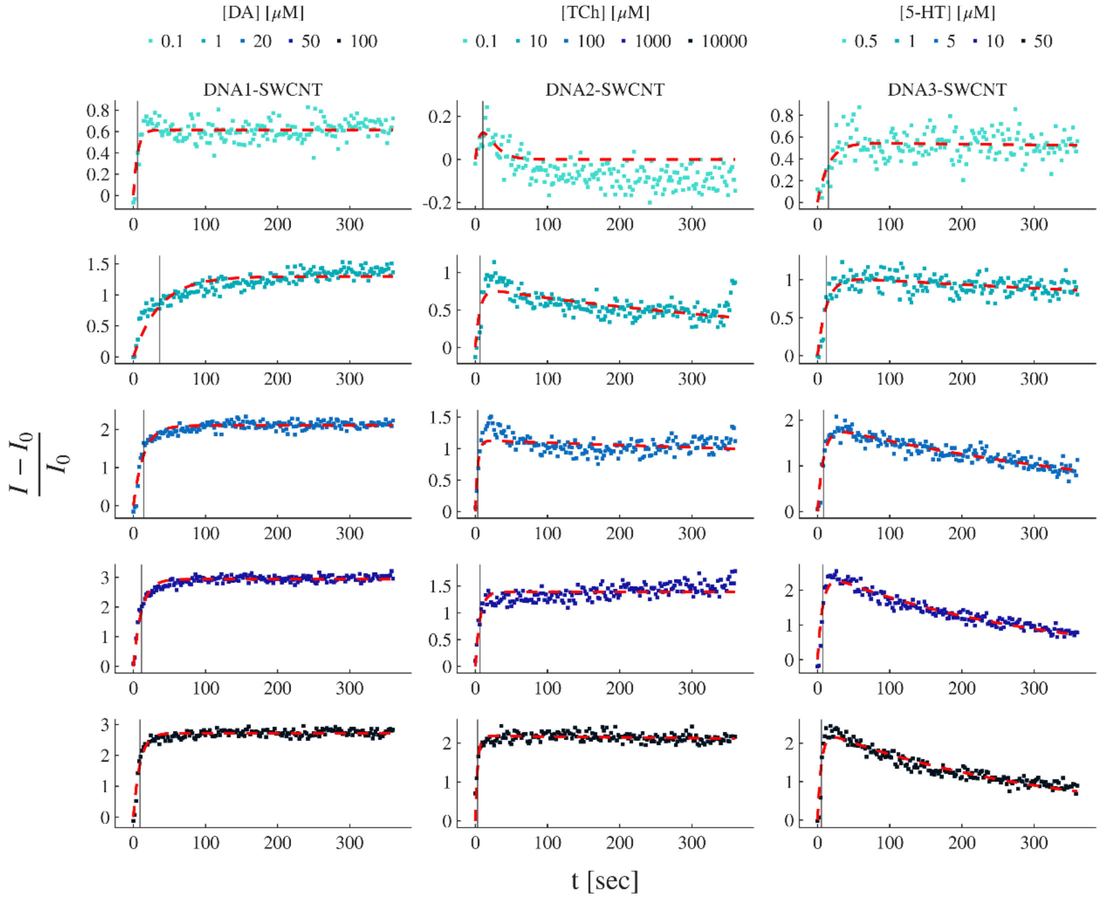

Figure S7. First cycle response data for DNA1-SWCNT with differing DA concentrations (left), DNA2-SWCNT with differing TCh concentrations (middle), DNA3-SWCNT with differing 5-HT concentrations (right), and their fitted difference of exponentials (dashed red line). The time constant of the increase in fluorescence ($1/k_r$) for each response is marked as a horizontal line.

Table S3. "Difference of exponentials" fit parameters of the first cycle response data from imaging experiments and their 95% confidence intervals. Responses with a k_d value of zero have only a fluorescence increase component.

	[Analyte] [µM]	A	k_d [s^{-1}] $\times 10^3$	k_r [s^{-1}] $\times 10^3$
**DNA1-SWCNT**	0.1	0.615 (0.60, 0.63)	0	174.660 (116.77, 232.55)
	1	1.297 (1.27, 1.32)	0	27.469 (24.72, 30.22)
	20	2.105 (2.09, 2.12)	0	69.885 (64.12, 75.65)
	50	2.941 (2.92, 2.96)	0	88.284 (82.48, 94.08)
	100	2.718 (2.70, 2.74)	0	109.143 (100.52, 117.76)
**DNA2-SWCNT**	10^{-1}	4.384 (0, 3207.59)	88.937 (0, 2559.97)	96.055 (0, 2834.28)
	10	0.802 (0.75, 0.86)	1.925 (1.55, 2.29)	153.675 (92.55, 214.80)
	10^2	1.132 (1.09, 1.17)	0.352 (0.17, 0.53)	297.821 (189.87, 405.77)
	10^3	1.392 (1.37, 1.41)	0	154.673 (121.47, 187.87)
	10^4	2.188 (2.15, 2.22)	0.091 (0.01, 0.17)	317.318 (261.86, 372.78)
**DNA3-SWCNT**	0.5	0.549 (0.50, 0.60)	0.130 (0, 0.52)	66.132 (41.44, 90.82)
	1	1.043 (1.00, 1.09)	0.534 (0.33, 0.73)	81.102 (65.15, 97.05)
	5	1.897 (1.84, 1.95)	2.056 (1.90, 2.21)	119.570 (102.27, 136.87)
	10	2.552 (2.48, 2.63)	3.524 (3.34, 3.71)	137.039 (118.01, 156.07)
	50	2.357 (2.29, 2.42)	3.186 (3.01, 3.36)	185.886 (155.71, 216.06)

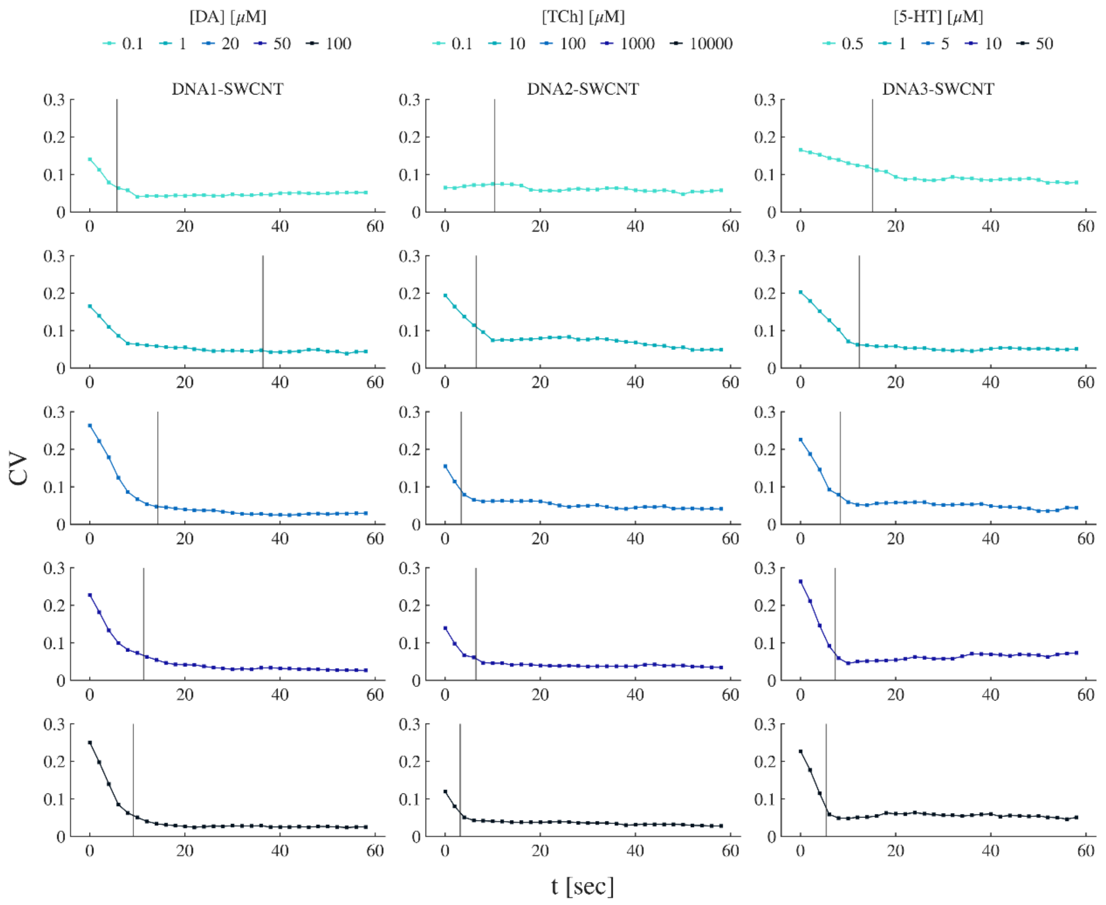

Figure S8. First cycle CV of I computed over 1-minute intervals starting at different times after the onset of the response for DNA1-SWCNT with different DA concentrations (left), DNA2-SWCNT with different TCh concentrations (middle), and DNA3-SWCNT with different 5-HT concentrations (right). The time constant corresponding to the increase in fluorescence ($1/k_r$) for each response is marked as a horizontal line.

`

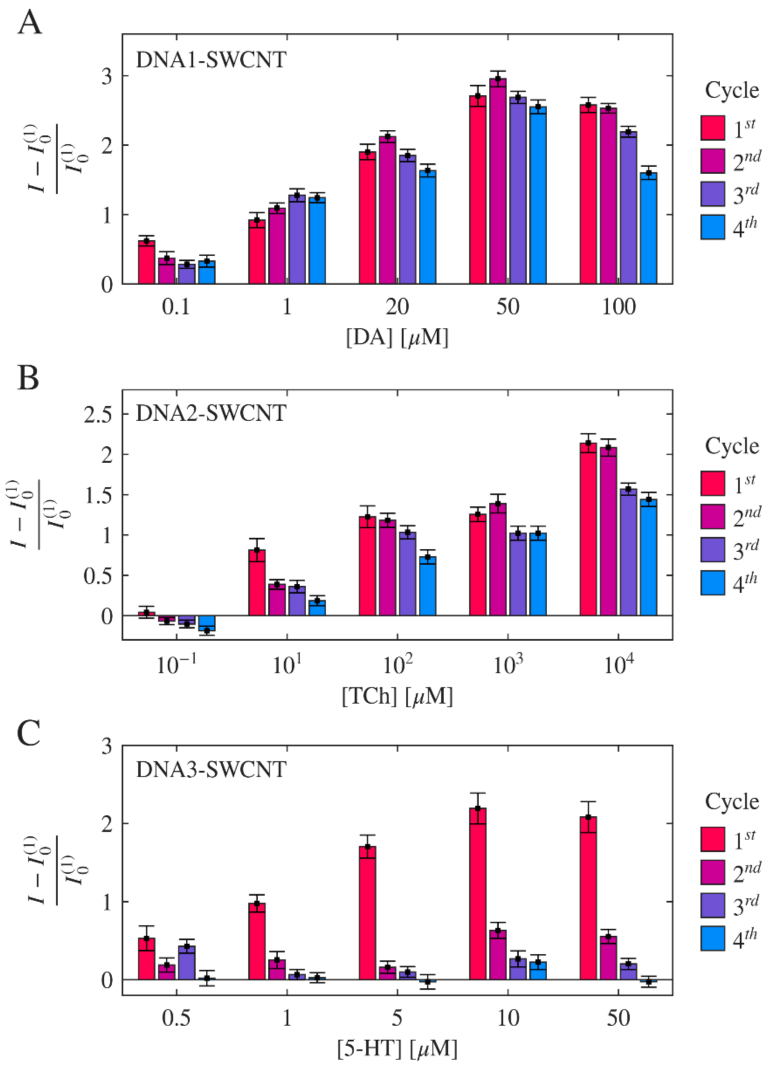

Figure S9. Normalized response of DNA-SWCNTs FOV for different analyte concentrations and cycles, where the normalized response is calculated with post-analyte fluorescence values from the current cycle, I, and baseline values from the first cycle, $I_0^{(1)}$. (A) DNA1-SWCNT with DA. (B) DNA2-SWCNT with TCh. (C) DNA3-SWCNT with 5-HT.

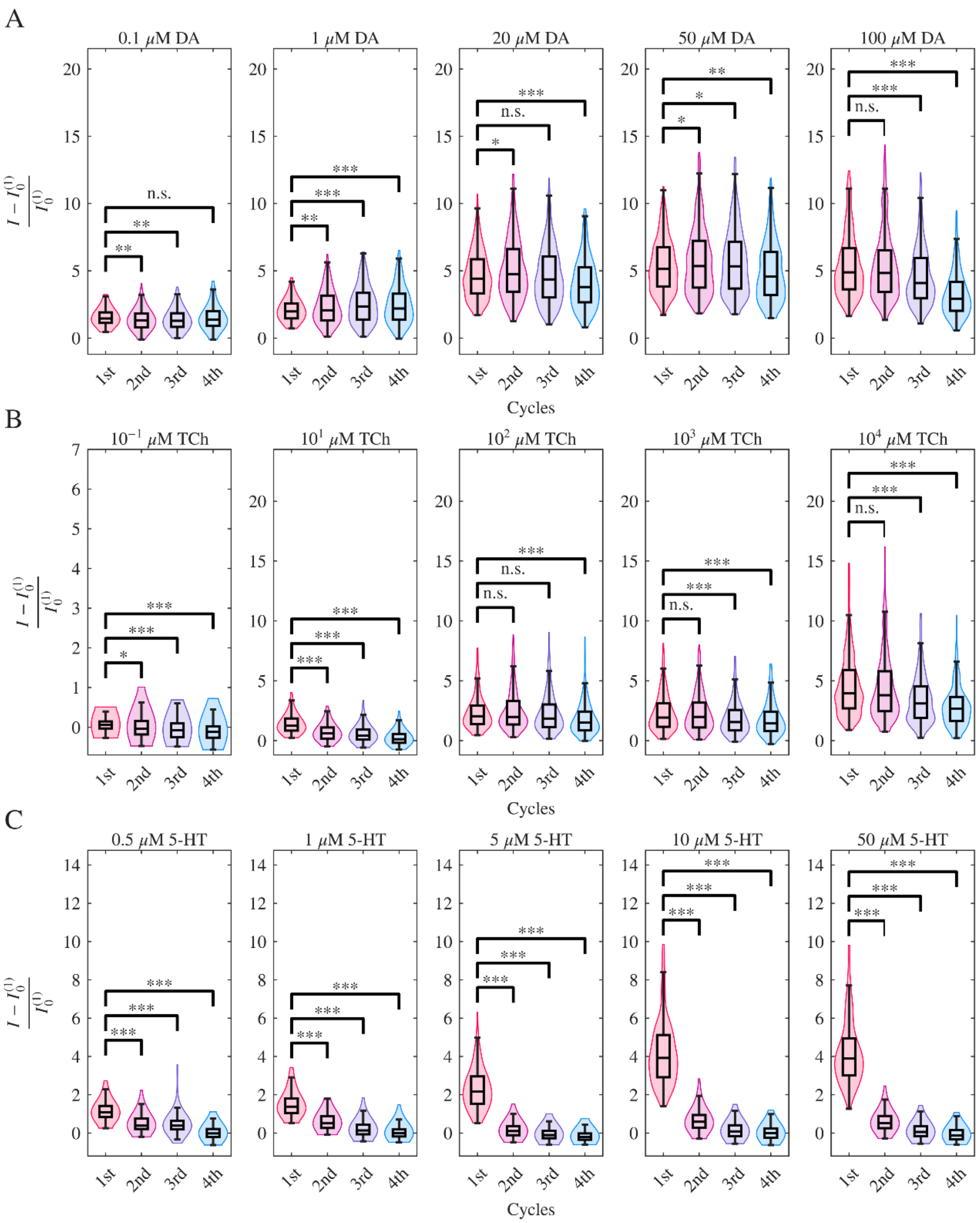

Figure S10. Distribution of the normalized fluorescence response $\left(I - I_0^{(1)}\right)/I_0^{(1)}$ to analyte through cycles in the segmented ROI population for each DNA-SWCNT and analyte concentration, where I was taken from the current cycle, and $I_0^{(1)}$ the first cycle. (A) DNA-SWCNT with DA as an analyte. (B) DNA2-SWCNT with TCh as an analyte. (C) DNA3-SWCNT with 5-HT as an analyte. Statistical significance was analyzed using one-way ANOVA tests, n.s. p>0.05, *p<0.05, **p < 0.01, ***p < 0.001.

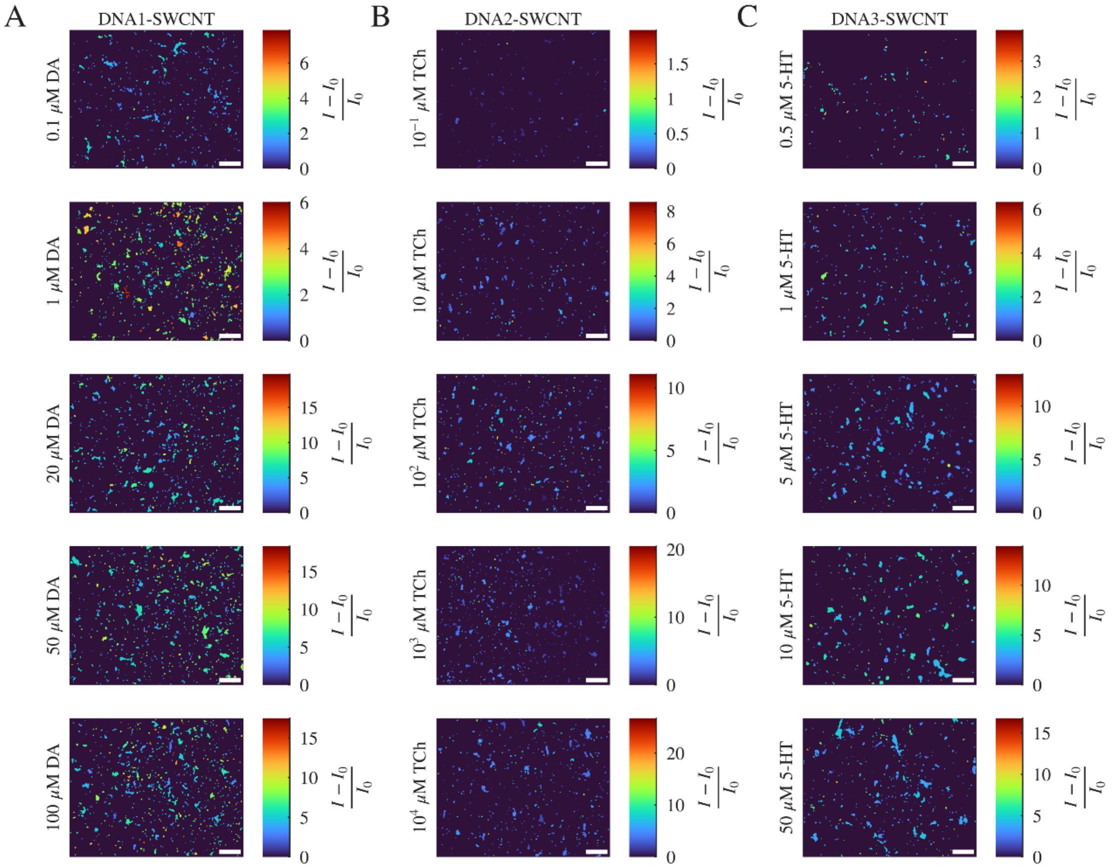

Figure S11. Normalized fluorescence response $(I - I_0)/I_0$ of individual ROIs in the first cycle of flow experiments with different analyte concentrations. (A) DNA1-SWCNT with DA as an analyte. (B) DNA2-SWCNT with TCh as an analyte. (C) DNA3-SWCNT with 5-HT as an analyte. Scale bar = 10 μm.

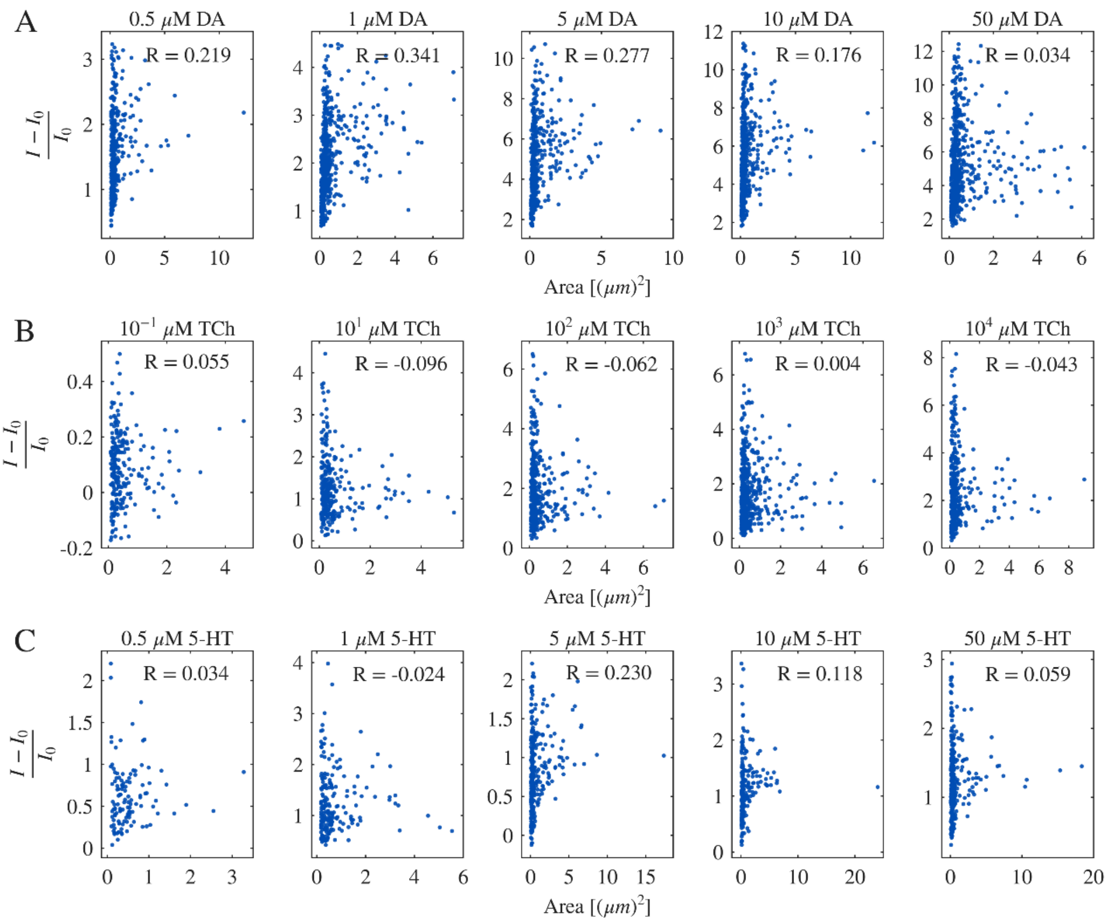

Figure S12. Individual ROI normalized fluorescence response $(I - I_0)/I_0$ vs. ROI area in the first cycle of flow experiments with different analyte concentrations. The Pearson coefficient, R, between the two variables was calculated to determine linear correlation. (A) DNA1-SWCNT with DA as analyte. (B) DNA2-SWCNT with TCh as analyte. (C) DNA3-SWCNT with 5-HT as analyte. Each dot represents a different ROI.

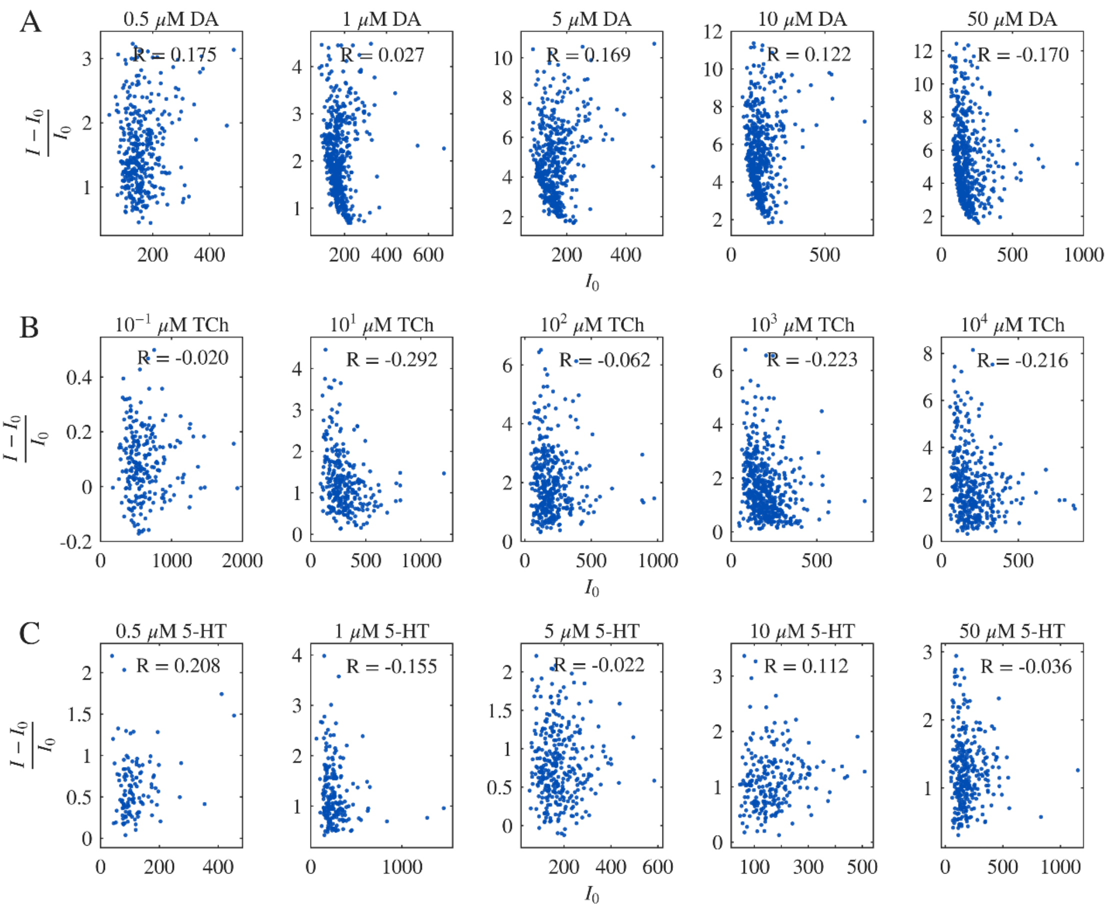

Figure S13. Individual ROI normalized fluorescence response $(I - I_0)/I_0$ vs. baseline fluorescence (I_0) in the first cycle of flow experiments with different analyte concentrations. The Pearson coefficient, R, between the two variables was calculated to determine linear correlation. (A) DNA1-SWCNT with DA as analyte. (B) DNA2-SWCNT with TCh as analyte. (C) DNA3-SWCNT with 5-HT as analyte. Each dot represents a different ROI.

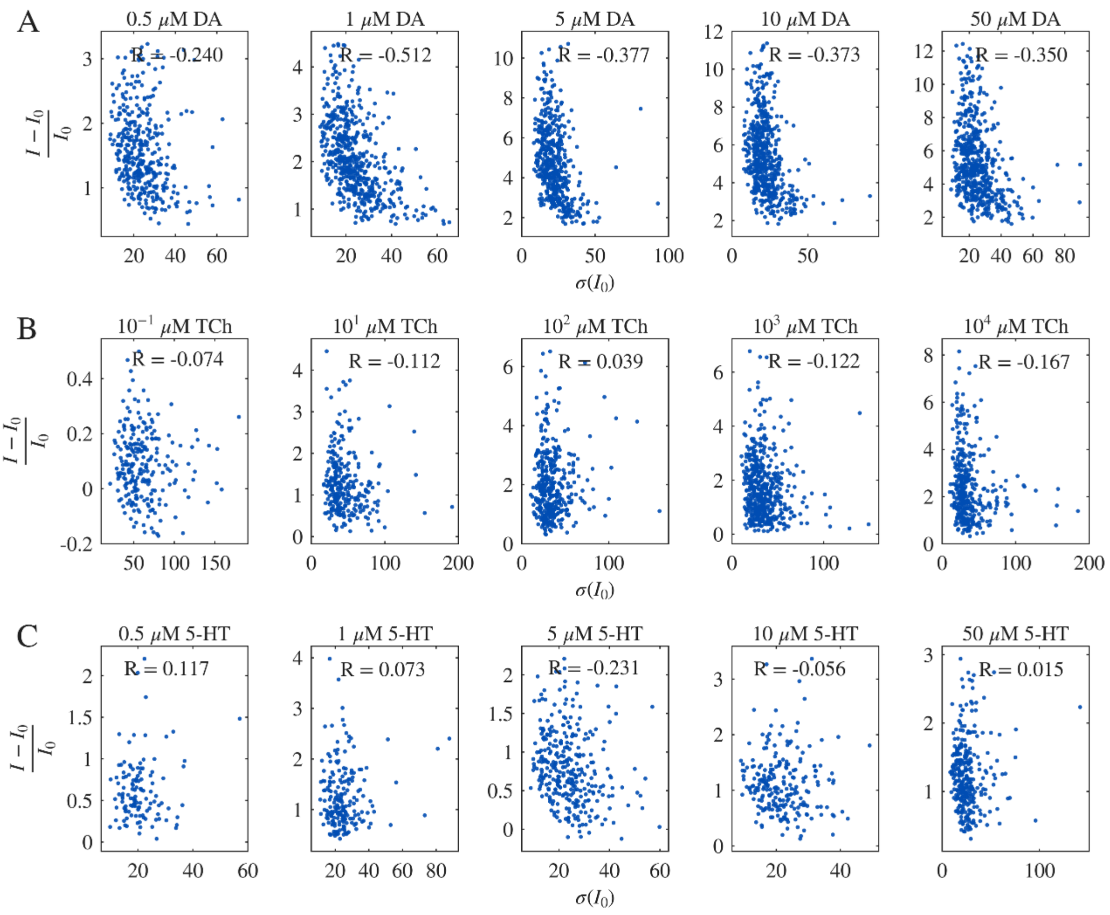

Figure S14. Individual ROI normalized fluorescence response $(I - I_0)/I_0$ vs. the standard deviation of baseline fluorescence ($\sigma(I_0)$) in the first cycle of flow experiments with different analyte concentrations. The Pearson coefficient, R, between the two variables was calculated to determine linear correlation. (A) DNA1-SWCNT with DA as analyte. (B) DNA2-SWCNT with TCh as analyte. (C) DNA3-SWCNT with 5-HT as analyte. Each dot represents a different ROI.

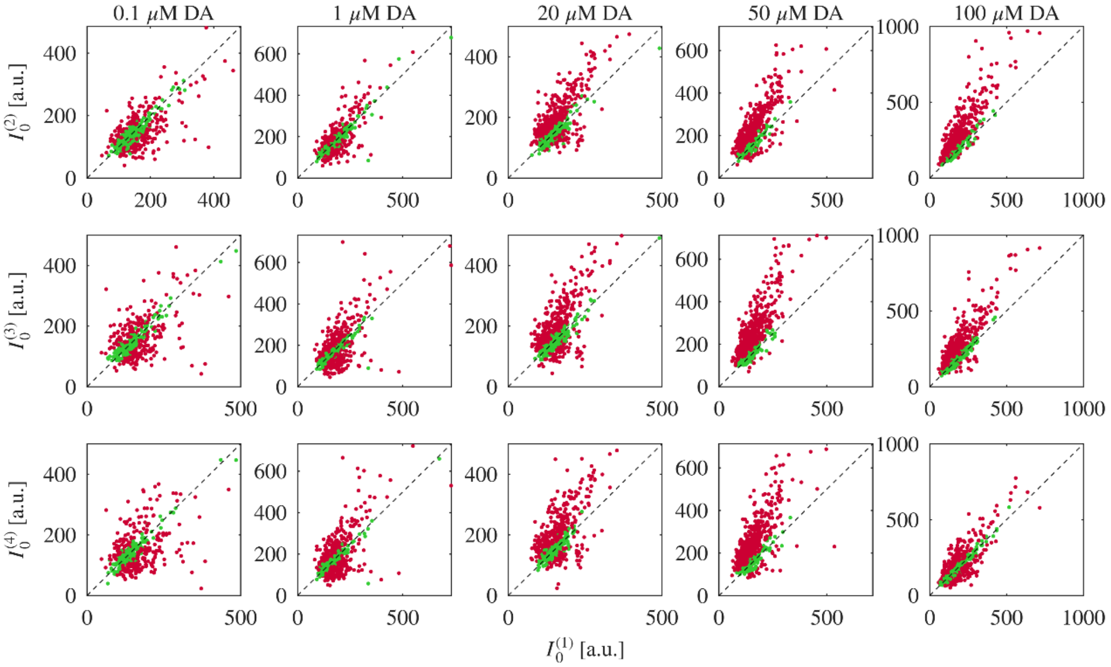

Figure S15. Comparison of I_0 across cycles for DNA1-SWCNT at varying DA concentrations. Each point is an individual ROI, plotting later-cycle I_0 (y-axis) versus first-cycle $I_0^{(1)}$ (x-axis). The dashed line denotes the $x = y$ line to aid visualization of increases (above the line) or decreases (below the line) in I_0 relative to the first cycle. Green points indicate ROIs whose I_0 shift is within ±1 STD of that ROI's initial baseline cycle $I_0^{(1)}$ (noise range), and red points exceed ±1 STD, indicating a baseline change beyond noise.

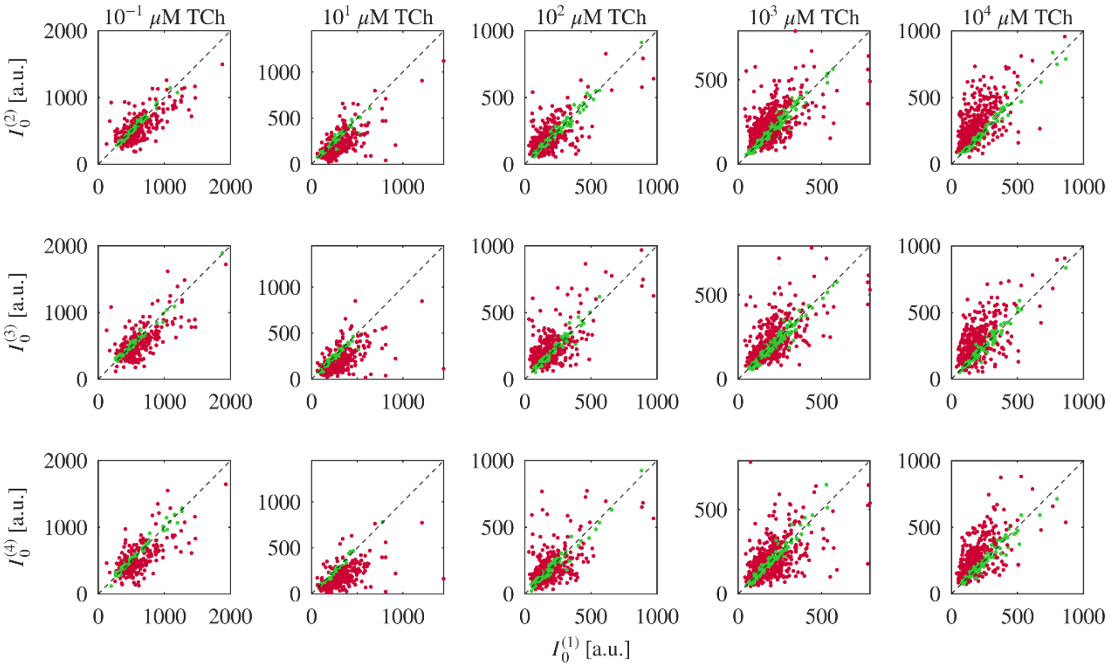

Figure S16. Comparison of I_0 across cycles for DNA2-SWCNT at varying TCh concentrations. Each point represents an individual ROI, plotting later-cycle I_0 (y-axis) versus first-cycle $I_0^{(1)}$ (x-axis). The dashed line denotes the $x = y$ line to aid visualization of increases (above the line) or decreases (below the line) in I_0 relative to the first cycle. Green points indicate ROIs whose I_0 shift is within ±1 STD of that ROI's initial baseline cycle $I_0^{(1)}$(noise range), and red points exceed ±1 STD, indicating a baseline change beyond noise.

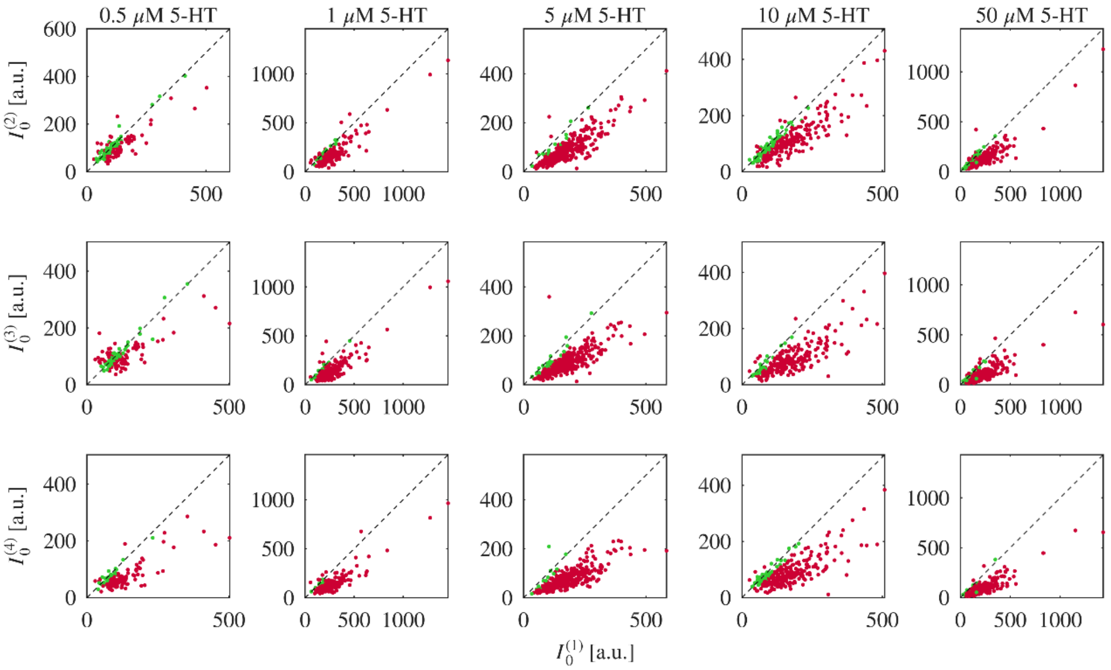

Figure S17. Comparison of I_0 across cycles for DNA3-SWCNT at varying 5-HT concentrations. Each point is an individual ROI, plotting later-cycle I_0 (y-axis) versus first-cycle $I_0^{(1)}$ (x-axis). The dashed line denotes the $x = y$ line to aid visualization of increases (above the line) or decreases (below the line) in I_0 relative to the first cycle. Green points indicate ROIs whose I_0 shift is within ±1 STD of that ROI's initial baseline cycle $I_0^{(1)}$ (noise range), and red points exceed ±1 STD, indicating a baseline change beyond noise.

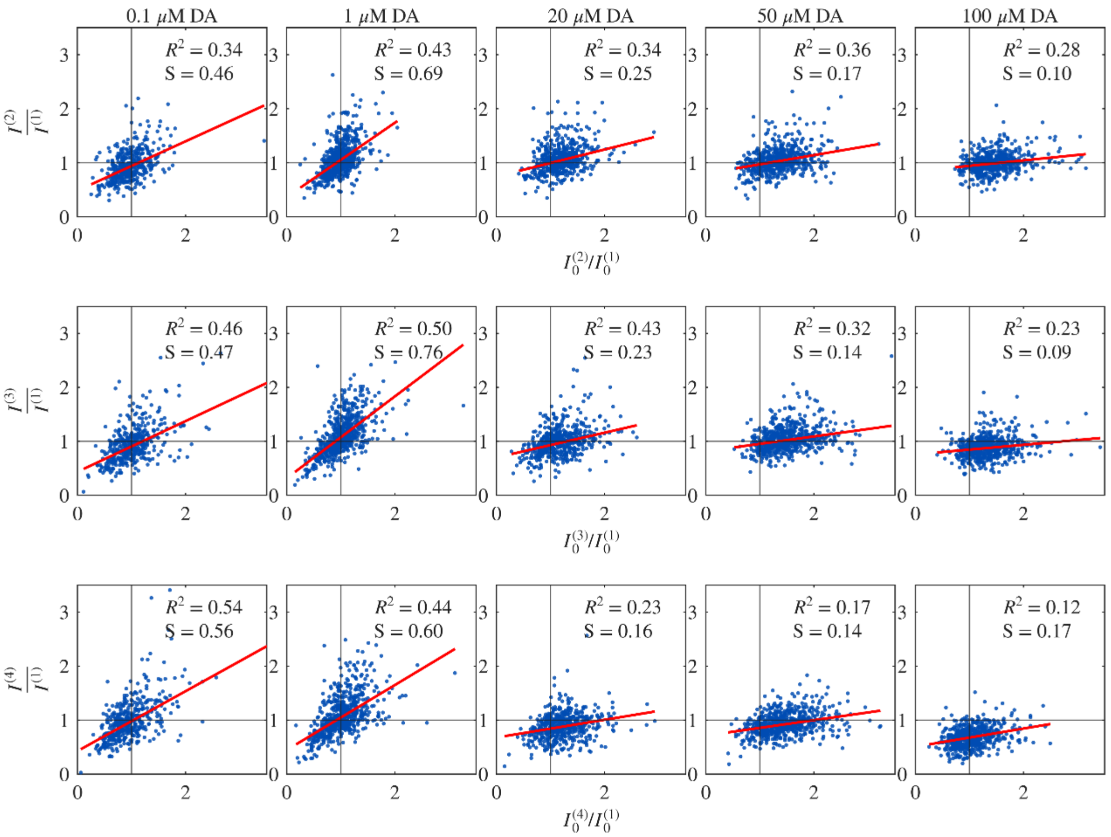

Figure S18. Comparison of the relative change in I values to the relative change in I_0 values between the first and later cycles, for individual ROIs of DNA1-SWCNT and varying DA concentrations. Each dot represents an individual ROI. The black lines represent the $x = 1$ and $y = 1$ lines as guides to the eye. The coefficient of determination (R^2) of the data in relation to a linear fit, and the fit's slope (S) are presented, as well as the fitted curve (red).

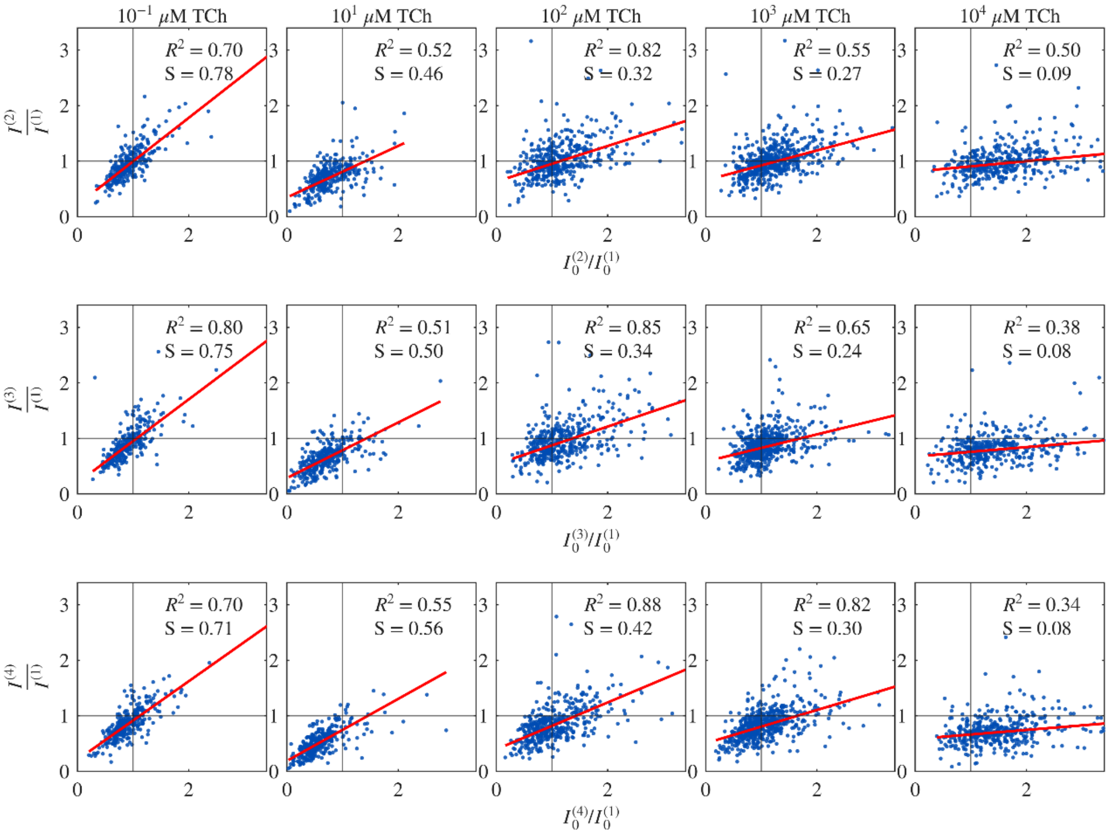

Figure S19. Comparison of the relative change in I values to the relative change in I_0 values between the first and later cycles, for individual ROIs of DNA2-SWCNT and varying TCh concentrations. Each dot represents an individual ROI. The black lines represent the $x = 1$ and $y = 1$ lines as guides to the eye. The coefficient of determination (R^2) of the data in relation to a linear fit, and the fit's slope (S) are presented, as well as the fitted curve (red).

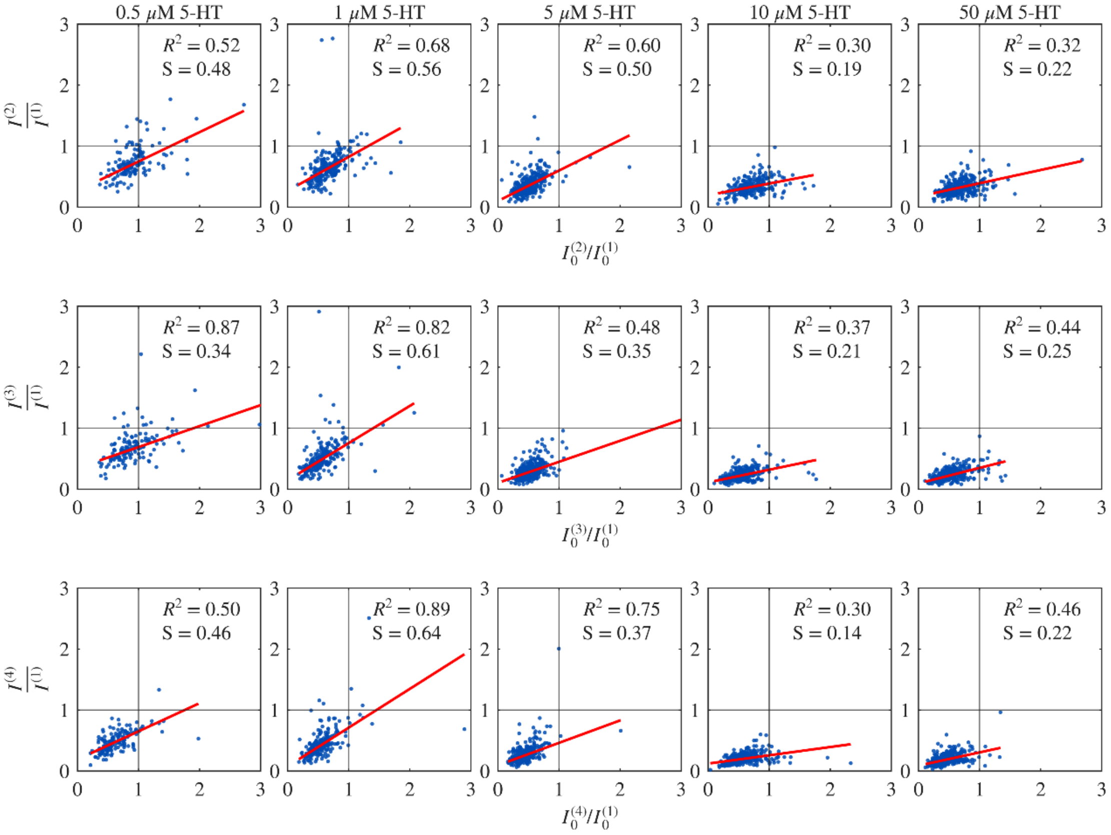

Figure S20. Comparison of the relative change in I values to the relative change in I_0 values between the first and later cycles, for individual ROIs of DNA3-SWCNT and varying 5-HT concentrations. Each dot represents an individual ROI. The black lines represent the $x = 1$ and $y = 1$ lines as guides to the eye. The coefficient of determination (R^2) of the data in relation to a linear fit, and the fit's slope (S) are presented, as well as the fitted curve (red).

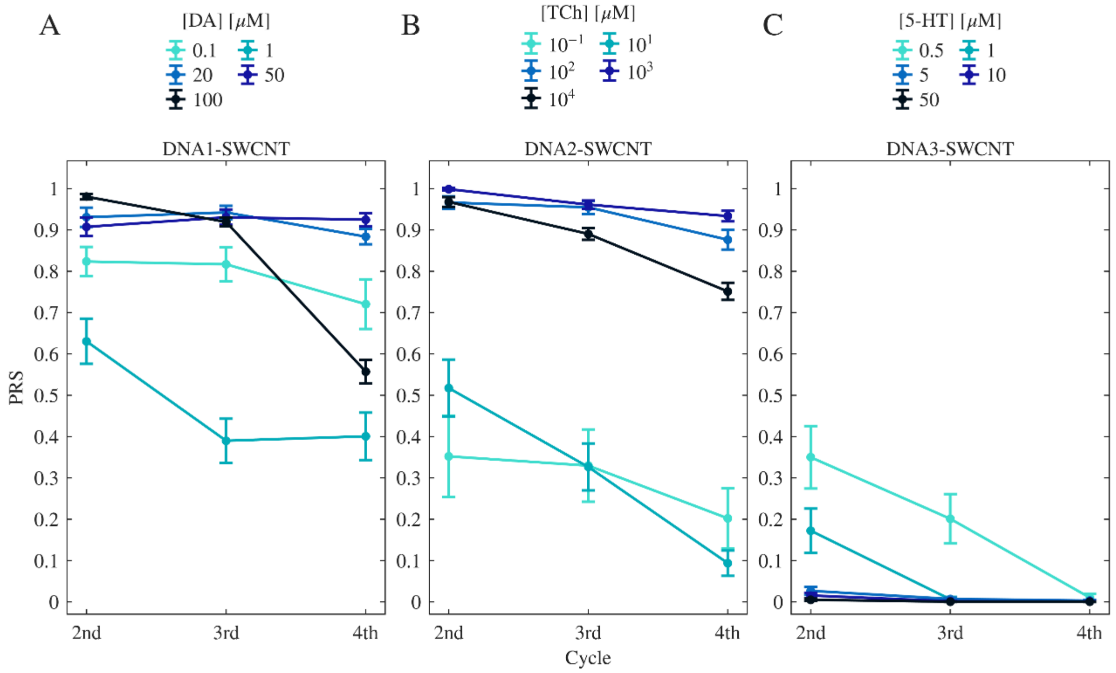

Figure S21. Reversibility Score of normalized fluorescence response, $(I - I_0)/I_0$, of the DNA-SWCNTs to varying analyte concentration across exposure cycles, using a fixed baseline $I_0^{(1)}$. From left to right: DNA1-SWCNTs with DA as an analyte, DNA2-SWCNT with TCh as an analyte, DNA3-SWCNT with 5-HT as an analyte.

List of Movies:

Movie S1: Flow Experiment of immobilized 0.5 mg L^{-1} DNA1-SWCNTs with 0.1 µM DA as an analyte. The video was taken with a 100X objective. Scale bar is 10 µm.

Movie S2: Flow Experiment of immobilized 0.5 mg L^{-1} DNA1-SWCNTs with 1 µM DA as an analyte. The video was taken with a 100X objective. Scale bar is 10 µm.

Movie S3: Flow Experiment of immobilized 0.5 mg L^{-1} DNA1-SWCNTs with 20 µM DA as an analyte. The video was taken with a 100X objective. Scale bar is 10 µm.

Movie S4: Flow Experiment of immobilized 0.5 mg L^{-1} DNA1-SWCNTs with 50 µM DA as an analyte. The video was taken with a 100X objective. Scale bar is 10 µm.

Movie S5: Flow Experiment of immobilized 0.5 mg L^{-1} DNA1-SWCNTs with 100 µM DA as an analyte. The video was taken with a 100X objective. Scale bar is 10 µm.

Movie S6: Flow Experiment of immobilized 0.5 mg L^{-1} DNA2-SWCNTs with 0.1 µM TCh as an analyte. The video was taken with a 100X objective. Scale bar is 10 µm.

Movie S7: Flow Experiment of immobilized 0.5 mg L^{-1} DNA2-SWCNTs with 10 µM TCh as an analyte. The video was taken with a 100X objective. Scale bar is 10 µm.

Movie S8: Flow Experiment of immobilized 0.5 mg L^{-1} DNA2-SWCNTs with 100 µM TCh as an analyte. The video was taken with a 100X objective. Scale bar is 10 µm.

Movie S9: Flow Experiment of immobilized 0.5 mg L^{-1} DNA2-SWCNTs with 10^3 µM TCh as an analyte. The video was taken with a 100X objective. Scale bar is 10 µm.

Movie S10: Flow Experiment of immobilized 0.5 mg L^{-1} DNA2-SWCNTs with 10^4 µM TCh as an analyte. The video was taken with a 100X objective. Scale bar is 10 µm.

Movie S11: Flow Experiment of immobilized 0.5 mg L^{-1} DNA3-SWCNTs with 0.5 µM 5-HT as an analyte. The video was taken with a 100X objective. Scale bar is 10 µm.

Movie S12: Flow Experiment of immobilized 0.5 mg L^{-1} DNA3-SWCNTs with 1 µM 5-HT as an analyte. The video was taken with a 100X objective. Scale bar is 10 µm.

Movie S13: Flow Experiment of immobilized 0.5 mg L^{-1} DNA3-SWCNTs with 5 µM 5-HT as an analyte. The video was taken with a 100X objective. Scale bar is 10 µm.

Movie S14: Flow Experiment of immobilized 0.5 mg L^{-1} DNA3-SWCNTs with 10 µM 5-HT as an analyte. The video was taken with a 100X objective. Scale bar is 10 µm.

Movie S15: Flow Experiment of immobilized 0.5 mg L^{-1} DNA3-SWCNTs with 50 µM 5-HT as an analyte. The video was taken with a 100X objective. Scale bar is 10 µm.

1. Kruss S, Landry MP, Vander Ende E, et al. Neurotransmitter Detection Using Corona Phase Molecular Recognition on Fluorescent Single-Walled Carbon Nanotube Sensors. *J Am Chem Soc*. 2014;136(2):713-724. doi:10.1021/ja410433b
